# Naming assessment in individuals with neurodegenerative diseases from different languages and cultures: a proposal

**DOI:** 10.1002/alz70858_099707

**Published:** 2025-12-24

**Authors:** Emelia Lázaro, Ana‐Inès Ansaldo, Yves Joanette

**Affiliations:** ^1^ Université de Montréal, Montréal, QC, Canada; ^2^ IUGM research center, Montréal, QC, Canada; ^3^ Canada Institutes of Health Research, Montreal, QC, Canada

## Abstract

**Background:**

It is well recognized that language difficulties can emerge in the early stages of dementia. Errors in naming, word‐finding difficulties, and the tip‐of‐the‐tongue phenomenon, among others, can serve as early indicators of neurodegenerative diseases. However, the assessment of naming is not always conducted comprehensively. This issue may be partly due to the limited number of available instruments that are sensitive enough to evaluate the psycholinguistic characteristics of stimuli in detail. For example, some studies have also highlighted the impact of semantic category or low agreement on naming in clinical populations, such as in Alzheimer's disease. Additionally, in intercultural contexts, the assessment becomes even more complex, as it faces challenges such as the use of instruments that are not always appropriately adapted when applied in a language or culture different from that in which the test was originally developed.

Given the need for more sensitive instruments in clinical populations with neurodegenerative diseases, and for these instruments to be interculturally relevant, the proposal aims to present a picture‐naming and action‐verb‐naming task, originally developed in French, that is part of the i‐MEL.fr protocol. This task is currently being adapted to non‐regional Spanish.

**Method:**

The adaptation methodology considers psycholinguistic aspects such as frequency, naming agreement, and confounding factors. The task includes 72 stimuli (48 nouns – from different semantic categories, both frequent and infrequent – and 24 action‐verbs, both transitive and intransitive), divided into 2 equivalent versions. To verify the relevance of the stimuli, healthy Spanish‐speaking participants from five different regions took part in the study (570 for nouns and 415 for verbs).

**Result:**

A detailed analysis identified 61 equivalent stimuli (39 nouns and 20 verbs), while the remaining 11 (9 nouns and 2 verbs) showed differences due to frequency, naming agreement, or other confounding factors, consequently, its change is suggested.

**Conclusion:**

The study highlights the importance of methodological rigor in adapting neuropsychological tools across languages and cultures. This approach is crucial for advancing the early identification of difficulties and improving the understanding and diagnosis of neurodegenerative diseases, especially in intercultural contexts where linguistic and cultural differences can affect assessment accuracy.

